# Cerebrospinal Fluid Proteomic Changes after Nusinersen in Patients with Spinal Muscular Atrophy

**DOI:** 10.3390/jcm12206696

**Published:** 2023-10-23

**Authors:** Marie Beaudin, Tahereh Kamali, Whitney Tang, Katharine A. Hagerman, Sally Dunaway Young, Lisa Ghiglieri, Dana M. Parker, Benoit Lehallier, Carolina Tesi-Rocha, Jacinda B. Sampson, Tina Duong, John W. Day

**Affiliations:** 1 Department of Neurology and Neurological Sciences, Stanford School of Medicine, Stanford, CA 94304, USAtkamali@stanford.edu (T.K.); whitneyt@stanford.edu (W.T.); khagerma@stanford.edu (K.A.H.); lehallib@stanford.edu (B.L.); ctesiroc@stanford.edu (C.T.-R.);; 2 Department of Neurology, Stanford Health Care, Stanford, CA 94304, USA

**Keywords:** spinal muscular atrophy, nusinersen, treatment, proteomics, biomarkers, neurofilament

## Abstract

Disease-modifying treatments have transformed the natural history of spinal muscular atrophy (SMA), but the cellular pathways altered by SMN restoration remain undefined and biomarkers cannot yet precisely predict treatment response. We performed an exploratory cerebrospinal fluid (CSF) proteomic study in a diverse sample of SMA patients treated with nusinersen to elucidate therapeutic pathways and identify predictors of motor improvement. Proteomic analyses were performed on CSF samples collected before treatment (T0) and at 6 months (T6) using an Olink panel to quantify 1113 peptides. A supervised machine learning approach was used to identify proteins that discriminated patients who improved functionally from those who did not after 2 years of treatment. A total of 49 SMA patients were included (10 type 1, 18 type 2, and 21 type 3), ranging in age from 3 months to 65 years. Most proteins showed a decrease in CSF concentration at T6. The machine learning algorithm identified ARSB, ENTPD2, NEFL, and IFI30 as the proteins most predictive of improvement. The machine learning model was able to predict motor improvement at 2 years with 79.6% accuracy. The results highlight the potential application of CSF biomarkers to predict motor improvement following SMA treatment. Validation in larger datasets is needed.

## 1. Introduction

Spinal muscular atrophy (SMA) is an autosomal recessive neuromuscular disease characterized by muscle weakness and wasting caused by degeneration of a-motor neurons in the brainstem and spinal cord. It is caused by homozygous deletions or compound heterozygous mutations in the survival motor neuron 1 (*SMN1*) gene. The incidence is estimated to vary significantly between countries and populations over a range of 1/6000 to 1/19,000 in newborn screening programs [1,2,3,4]. The phenotype is modified by the presence of a paralogous gene, *SMN2*, in which alternative splicing typically excludes exon 7, resulting in a non-functional protein that is rapidly degraded. Approximately 10% of *SMN2* transcripts are of the full length required to generate functional SMN protein [5,6], and the number of *SMN2* copies is the major genetic modifier of disease severity [7]. Traditionally, SMA patients have been classified according to age at onset and maximal functional level achieved. SMA type 1 is characterized by onset in the first 6 months of life and a median survival without respiratory support of 13.5 months before the development of disease-modifying therapies [8]. SMA type 2 becomes clinically manifest between 6 and 18 months of age, and patients typically gain the ability to sit, but not to walk. Patients with SMA type 3 have symptom onset after 18 months of age and achieve independent walking, although may lose this ability over time. Finally, SMA type 4 patients are at the mildest end of the spectrum and develop weakness in adulthood after normal motor development and function.

The natural history of SMA has been transformed by the approval of disease-modifying therapies, starting with nusinersen, a synthetic antisense oligonucleotide that alters the splicing of *SMN2* pre-mRNA and promotes the production of functional SMN protein [9]. The approval was based on the results of the ENDEAR study [10], which showed improvement in motor milestones in 51% of treated SMA type 1 patients. This was followed by subsequent studies showing positive results in patients with other SMA types and ages [11,12]. Clinical response is variable, with 25–50% of type 2 or type 3 patients showing clinically meaningful motor improvement at 6 months, while a larger group of patients have small improvements or stable course that distinguishes them from the decline expected in untreated patients [13,14,15,16]. A minority of patients continue to lose function despite treatment, though possibly less rapidly than their natural history, but the factors underlying this range in treatment responses are not yet well understood [12,14]. Shorter disease duration and better baseline function prior to treatment are associated with better outcomes, with the best outcomes having been reported in very young patients treated in the presymptomatic stage [10,17,18]. This underscores the importance of timely diagnosis, but also the emerging need for predictive tools to monitor disease activity and response to treatment, and to identify which patients are likely to respond to additional treatment in order to reduce motor neuron loss and optimize outcomes [19].

Cerebrospinal fluid (CSF) proteomics provides an opportunity to better understand the pathways by which nusinersen affects SMA disease progression and identify early biomarkers that reflect disease activity and treatment effects. Because CSF is in direct contact with the degenerating ventral horn motor neurons affected by SMA, proteomic changes might parallel disease progression and response to treatment. Among other proteins, neurofilament light chain (NEFL) and phosphorylated neurofilament heavy chain (pNfH) are promising candidates [20]. Neurofilaments are neuronal cytoskeletal proteins that leak into CSF and blood with neuronal damage and axonal loss. They are useful biomarkers of disease activity in many neurological disorders [20], so are not specific to any single disease. In SMA patients, pNfH levels are elevated compared to healthy controls and decrease rapidly after starting nusinersen [21]. Lower absolute levels after the loading dose period correlated with better motor outcomes in a study of pre-symptomatically treated infants [17]. In older patients, the association is less clear, and studies have shown no clear correlation between neurofilament levels and motor response to treatment [22,23,24,25]. Recently published studies have identified some promising CSF biomarkers of nusinersen treatment response in SMA, notably cathepsin D [26], protein combinations [27], or muscle microRNAs [28]. These studies highlight the enormous potential of CSF biomarkers to predict motor response to treatment. However, larger studies covering the range of ages, SMA types, and functional levels seen in clinical practice are needed to better characterize CSF proteomic changes and identify informative biomarkers that can be used in clinical practice.

We conducted an exploratory proteomic study of CSF protein changes following nusinersen treatment to identify unexpected proteins and pathways implicated in treatment response in a diverse cohort of patients with SMA. We then used a supervised machine learning random forest algorithm to identify top proteins associated with motor improvement and develop a predictive model of motor improvement after 2 years of treatment using early proteomic changes and baseline clinical data.

## 2. Materials and Methods

### 2.1. Study Design and Population

We performed a retrospective analysis of CSF proteomic data and motor outcomes in patients who started nusinersen treatment. A convenience sample of pediatric and adult patients with genetically confirmed 5q SMA who started treatment with nusinersen between 2017 and 2018 and who had deposited CSF samples at baseline (T0) and 6 months (T6) in the Stanford Neuromuscular Repository was recruited for the study. Patients were treated at Stanford Medicine (Lucile Packard Children’s Hospital for pediatric patients and Stanford Health Care for adult patients) in Palo Alto, CA, USA.

Written informed consent was obtained from all participants or parents before inclusion. This study was conducted in compliance with the Declaration of Helsinki (Version 2013) and with other applicable regulatory requirements. The protocol was approved by the Ethics Committee of Stanford Health Care and Stanford Children’s Health Stanford University, Protocol #23888, initially approved 15 May 2012, updated 2023.

### 2.2. CSF Sampling and Proteomic Analysis

CSF was sampled immediately before the first intrathecal nusinersen injection and subsequently before each injection, according to the recommended loading doses at day 1, day 15, day 29, day 64, and subsequently every 4 months. The first 0.5 mL of each CSF collection was sent for cell counts and basic biochemistry, while the remaining CSF was centrifuged (1000 rpm for 15 min at 4 °C) to pellet all cellular material. Aliquots of supernatant were placed in cryovials that were then frozen at −80 °C until being used for proteomic analyses. A pilot study of 12 patients at multiple time points throughout the first year of treatment revealed most protein changes were evident before the 6-month time point, so we selected this time point for this main analysis (data presented in [29]).

Proteomic sample analysis was conducted using the Olink^®^ (Uppsala, Sweden) platform and reported in the Normalized Protein eXpression scale (NPX, Olink’s arbitrary quantifying unit which is in the Log2 scale). The Olink complete panel and Neuro Exploratory panel were used for this purpose, which, when these studies were carried out, characterized a total of 1113 peptides. Protein levels were measured by two analyte-specific DNA-tagged antibodies, Proseek probes, which were allowed to pair-wise bind to the target protein in the sample. A new PCR target sequence was then formed by a proximity-dependent DNA polymerization event, with subsequent amplification in a proximity extension assay. The resulting sequence was then detected and quantified using standard real-time PCR. Protein levels were normalized using the Intensity Normalized (v2) procedure by Olink, except for data from the Neuro Exploratory panel, which was normalized using the Inter-plate Controls (IPC) normalized procedure because of its bimodal distribution. For quality control, four Olink internal controls were added to each sample to monitor the quality of assay performance and the quality of the sample. This was conducted in two steps: (1) each sample was evaluated on the standard deviation (SD) of the internal controls, which was verified to be below 0.2 NPX; and (2) the deviation of the internal control concentration in each sample in comparison with the median value was verified to be less than 0.3 NPX.

### 2.3. Evaluation of Motor Improvement

Motor function was evaluated at baseline and repeatedly during clinical follow-up by physical therapists with expertise in SMA outcome measures. The Children’s Hospital of Philadelphia Infant Test of Neuromuscular Disorders (CHOP-initiation of treatment were used as the baseline, preferentially using assessments INTEND) [10], the Hammersmith Functional Motor Scale Expanded (HFMSE) [30,31], the Revised Upper Limb Module (RULM) [32], and/or the 6-Minute Walking Test (6MWT) [33,34,35,36] were performed based on functional ability. Functional assessments are conducted closest to prior to the start of treatment to enhance the detection of early improvement. Evaluation of motor outcome was performed after two years of treatment or at last clinical follow-up for patients followed in our clinic for less than two years.

In many SMA patients, nusinersen therapeutic benefits are documented by slowing or halting their rate of motor decline, rather than by improved function, making it impossible to differentiate “responders” from “non-responders”. Consequently, patients were categorized into those whose motor function improved relative to the natural history (subsequently referred to as “improvers”) vs. those whose course declined or followed natural history trajectory (“non-improvers”), as determined by a decisional algorithm (see Appendix A). This algorithm accounted for the diversity of ages and baseline motor functions of the SMA patients included in this study. In brief, outcomes on the CHOP-INTEND were prioritized for all SMA type 1 patients [8,37,38,39] and all patients under 24 months of age. HFMSE was prioritized for type 2 and type 3 non-ambulatory patients, while 6MWT was prioritized for ambulant type 3 patients. RULM was used as secondary verification in type 2 and 3 patients. Motor improvement was assessed at the evaluation carried out closest to two years after starting treatment. Previously published minimal clinically important differences (MCIDs) and minimal detectable changes (MDCs) were used as cut-offs to determine whether individual patients had motor improvement. For patients that did not show significant change per published criteria, we defined age- and type-specific cut-offs based on natural history studies or progression patterns in placebo groups of large clinical trials [40,41,42,43,44,45,46,47,48] (see Appendix A for further details).

### 2.4. Statistical Analysis

Patients’ demographic and clinical characteristics are presented as percentages for categorical variables and means or medians according to the underlying distribution for continuous variables.

Proteomic changes between T0 and T6 were assessed for the whole group. Fold change was calculated for each protein and each patient as the ratio of concentrationT6/concentrationT0 using raw data. The *p*-values were calculated using the paired *t*-test. Visual analysis of the proteomic data was performed using volcano graphs, which allowed proteins with significant changes at T6 to be identified in the whole cohort. Gene ontology and pathway analysis were performed to identify the cellular pathways affected by the treatment. To do this, we extracted the significantly increased or decreased proteins using *p*-value < 0.05 and fold change and ran a pathway enrichment analysis with Gprofiler, using all proteins as the background.

To identify the top proteins predictive of motor improvement, a supervised random forest machine learning algorithm was implemented. The input to the random forest algorithm [29] consisted of the relative changes of the previously identified significantly altered proteins. The relative changes were calculated as (fold change −1) × 100 and the values were normalized. Each protein served as a distinct feature within the model, with each patient possessing a corresponding value representing the specific change in protein expression. Utilizing an ensemble of decision trees, the random forest method assessed the importance of each feature through an evaluation of the reduction in Gini impurity or entropy at each decision node. By averaging these reductions across all trees within the forest, the algorithm ascertained an importance score for each protein, thereby quantifying its contribution to the model’s predictive capacity. These importance scores were then employed to select the top proteins that were most predictive of motor improvement after treatment.

Mean and median NPX values of these top proteins were compared between T0 and T6 for improvers and non-improvers separately. Individual patient-level NPX values were plotted to assess for interindividual fluctuations. Patients were ranked from youngest to oldest to visually assess the effect of age. Cathepsin D (CSTD) was selected a priori to be assessed in the same way given its previous identification as a potential biomarker of nusinersen treatment response [26]. Mean relative concentration changes were compared between motor improvers and non-improvers with the use of a *t*-test.

The same random forest algorithm was used as a predictive model of motor improvement at 2 years using the relative change of the selected proteins and baseline clinical data. Clinical variables included were SMA type, age at onset, age at treatment initiation, sex, and functional status [15]. Leave-one-out cross-validation was performed on this model whereby model learning was conducted with n-1 samples and tested on the remaining sample, yielding a predicted success probability for each sample. This procedure was repeated *n* times. Receiver operating characteristic (ROC) curves were created by plotting sensitivity (or true positives) against 1-specificity (false positives) when each predicted success probability value is taken as a cutoff for binary classification. The area under the curve (AUC) and its respective *p*-value were calculated for ROC curves, given the null hypothesis of AUC = 0.5.

## 3. Results

A collection of 49 patients was included in the analysis, of which 10 were type 1, 18 were type 2, and 21 were type 3. Baseline motor assessments were performed at a median of 0 days before the start of nusinersen treatment (interquartile range (IQR) −75 to +105 days) and final motor assessments at a median of 746 days after the start of treatment (IQR 610 to 890 days). Demographic and baseline clinical characteristics are presented in Table 1. A total of 31 patients were determined to have shown functional improvement based on the motor improvement algorithm, with a higher proportion in the youngest and most seriously affected patients (type 1) compared to the older and less severely affected patients (types 2 and 3). Results on standardized motor scales at baseline and after two years of nusinersen treatment are presented in Table 2. More patients who showed functional improvement were captured with the CHOP-INTEND, as expected, given the larger proportion of patients with SMA type 1 who improved compared with the other SMA types. Many patients had more than one scale performed during clinical follow-up.

### 3.1. CSF Proteomic Changes after 6 Months of Nusinersen Treatment in the Whole Cohort

**Figure 1:**
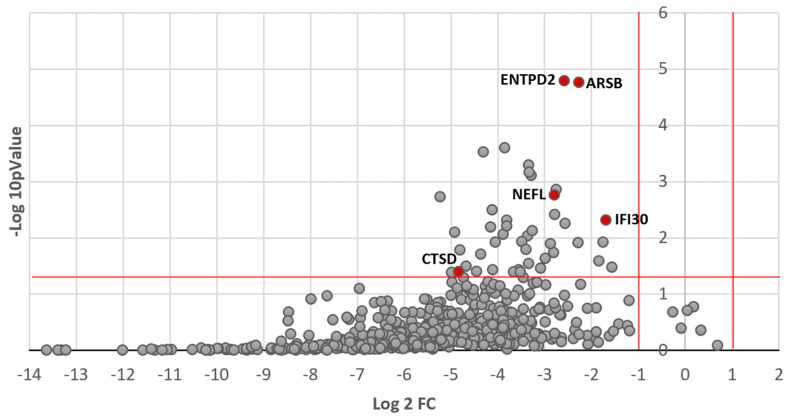
Volcano plot of exploratory proteomic analysis showing CSF concentration changes between T0 and T6 for the whole cohort. Log2 of the fold change is represented on the x-axis and -log10 of the *p* value on the y-axis. The horizontal red line indicates *p* = 0.05 and the vertical red lines indicate a fold change greater than 1 or inferior to −1. Most samples with statistically significant *p*-values are clustered between −1 and −5 on the x-axis, which indicates a significant decrease (at least 0.5-fold change) in the concentration of these proteins. Top predictive proteins and CTSD are identified on the graph for reference, and all 43 significantly altered peptides are listed in the supplementary table.

Overall, 595 out of the 1113 tested peptides were reliably detected. Figure 1 shows the volcano plot of concentration changes for the 595 detected proteins in the whole cohort. As depicted, most proteins showed a reduction in their CSF concentration after 6 months of treatment with nusinersen, with 43 proteins showing a statistically significant reduction (see Supplementary Table S1). Gene ontology and pathway analysis identified that factors that significantly changed after treatment were linked to neuronal processes/CNS. KEGG analysis suggested an effect on lysosomal function.

### 3.2. CSF Proteomic Profile Differs for Patients Demonstrating Motor Improvement

**Figure 2:**
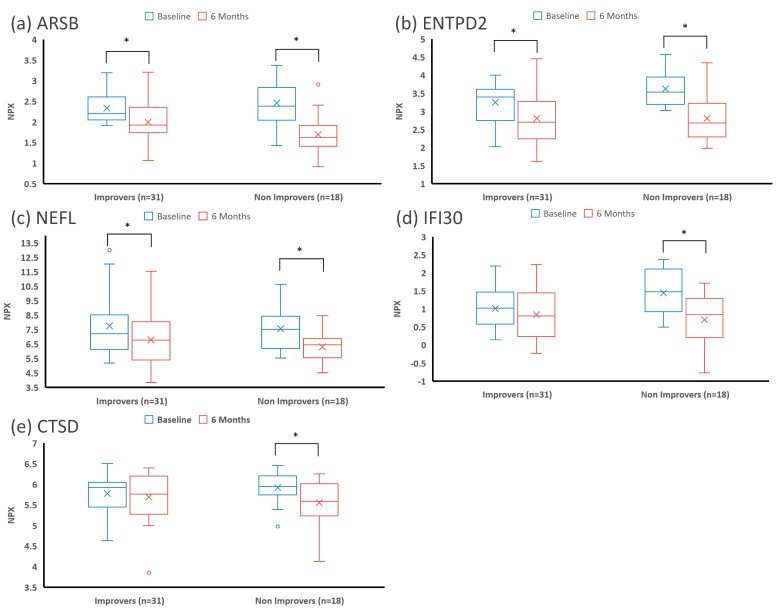
Boxplots of the CSF concentrations of the top proteins and CTSD at baseline and after 6 months of nusinersen treatment in patients showing motor improvement vs. no motor improvement. First quartile, median, and third quartile are marked with horizontal lines, mean is marked with an X, and data spread is shown with the whiskers (1.5*IQR, adjusted for skewness). Concentrations of (**a**) ARSB, (**b**) ENTPD2, (**c**) NEFL, (**d**) IFI30, and (**e**) CSTD are represented. All these proteins decreased after nusinersen treatment. Statistically significant differences (*p* < 0.05) are marked with a *.

The random forest machine learning algorithm was used to identify the top proteins whose variation in concentration between T0 and T6 was most predictive of motor improvement at 2 years. Four proteins were identified by this process: arylsulfatase B (ARSB, also N-acetylgalactosamine-4-sulfatase), ectonucleoside triphosphate diphosphohydrolase 2 (ENTPD2), neurofilament light chain (NEFL), and interferon-gamma-inducible protein 30 (IFI30). As shown in Figure 1, these proteins had a significant decrease in their CSF concentration in the whole cohort. CSF concentrations of the top proteins and CTSD at T0 and T6 in patients who demonstrated motor improvement vs. those who did not are presented in Figure 2. While the average CSF concentration of these proteins decreased after nusinersen treatment, the decrease appeared to be less in those who demonstrated functional gain (Figure 2). However, there was no statistically significant difference between the decrease in CSF concentration for any individual protein when comparing improvers and non-improvers.

There was considerable interindividual variability with most patients showing a decrease, but some patients showing an increase in CSF peptide concentrations (see Figure S1 for individual patient-level changes). Visual assessment of Supplementary Figure S1 revealed that younger patients appeared to have a more significant decrease in NEFL levels compared with older patients, which was true in improvers and non-improvers. This effect of age was not seen for the other proteins.

### 3.3. Predictive Model of Functional Improvement Using CSF Proteomic Changes

**Figure 3:**
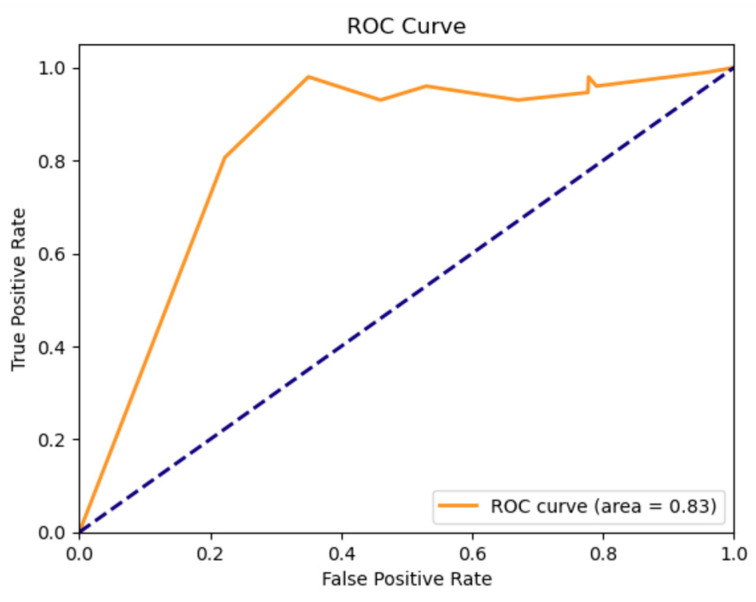
ROC curve of the machine learning predictive model of motor improvement at 2 years, depicting true positive rate (sensitivity) on the y axis and false positive rate (1-specificity) on the x-axis. The Random Forest model ROC AUC was 0.83, showing a strong discriminative ability. The blue dotted line represents a non-discriminatory (random) model.

The random forest machine learning model could predict motor improvement at 2 years using baseline clinical data and CSF proteomic changes at 6 months with 79.6% accuracy. Sensitivity was 80.6% and specificity was 77.8%. The ROC curve is presented in Figure 3. The ROC AUC value was 0.83, denoting a strong discriminative capability of the model.

## 4. Discussion

This exploratory analysis of CSF proteomic profiles of SMA patients shows that most captured proteins show a significant decrease in concentration after 6 months of nusinersen treatment. We used a supervised machine learning algorithm to identify top proteins whose change in concentration between T0 and T6 had the highest predictive value for motor improvement at 2 years, and identified ARSB, ENTPD2, NEFL, and IFI30, although none of these proteins individually was significantly associated with motor improvement. The supervised random forest machine learning predictive model could predict with 79.6% accuracy the functional improvement at two years using CSF proteomic changes at 6 months and baseline clinical data.

In our study, NEFL concentration was significantly decreased in the whole cohort after 6 months of treatment, and NEFL was identified within the 4 top proteins with the highest predictive ability for motor improvement at 2 years. Neurofilaments have received considerable interest as markers of disease severity and treatment response in SMA and other neurological disorders [20]. Phosphorylated neurofilament-heavy chain (pNfH) was shown to be strongly elevated in symptomatic infants and children with SMA in comparison with healthy controls, and levels dropped rapidly after starting nusinersen [21]. In infants with SMA treated pre-symptomatically, higher baseline plasma pNfH levels correlated with more severe disease courses once the infants became symptomatic. Lower absolute levels after the loading dose period correlated with better motor outcomes in the NURTURE study [17]. Longitudinal studies showed that CSF and serum NEFL concentration were reduced and negatively correlated with motor response in patients with SMA type 1 (with 2 copies of SMN2) or in type 2 patients with short disease evolution (<6 months in one study), but not in type 2 patients with longer disease evolution [22,49,50]. In older patients, neurofilaments were not significantly elevated at baseline in comparison with controls [23,24,49]. Baseline NEFL was correlated with motor function in pediatric and adult type 2 and type 3 SMA patients, but there was no significant change following Nusinersen treatment [23,51]. Other studies have shown a mild but significant decrease in pNfH or NEFL after 6 months of treatment in adult SMA patients, but this did not correlate significantly with motor response [24,25]. In our study, we observed a significant reduction in NEFL CSF levels after 6 months of treatment that contributed to the prediction of motor improvement. It is possible that this effect was mainly driven by younger patients since visual assessment showed that the decrease in NEFL was more significant in younger patients, although this was present both in improvers and non-improvers. NFH and pNfH were not included in the Olink panel and therefore not evaluated in the current study.

In addition to NEFL, the random forest machine learning algorithm identified 3 other top proteins whose change in CSF concentration between baseline and 6 months was most predictive of functional improvement. These were ARSB, a lysosomal protein, ENTDP2, an ecto-ATPase involved in regulating purinergic neurotransmission, and IFI-30, a lysosomal thiol reductase involved in antigen presentation. ARSB has long been known to localize to lysosomes, and mutations in the ARSB gene cause mucopolysaccharidosis VI. More recent studies indicate a broader distribution at the cell membrane and nucleus with functions in tumor suppression, transcriptional mediation, redox balance, and cell signaling [52]. In a recent study of the SOD1-ALS mouse model, this protein was shown to be predominantly expressed in anterior horn cells of the spinal cord, and abnormal expression and distribution of ARSB was closely associated with neuron death, suggesting a role motor neuron cell death [53]. ENTPD2 is a membrane-bound extracellular enzyme that can hydrolyze ATP and other nucleotides to regulate purinergic neurotransmission. It is involved in enteric nervous system modulation of gut inflammation [54] and taste bud function [55], and was shown to be the major ectonucleotidase in rat astrocytes [56]. IFI30 is involved in MHC class II-restricted antigen processing, cross-presentation on MHC class I, modulation of proteolytic efficiency and degradation of lysosomal Cathepsin S, inhibition of T cell activation, reduction of cell proliferation, and reduction of autophagy [57]. Hence, this protein has been implicated in modulating autoimmunity and cancer survival.

Proteomic changes following nusinersen treatment have been studied by others [58]. A recent study using exploratory proteomic analysis in SMA type 1 patients showed that cathepsin D (CTSD), a lysosomal aspartyl protease, was down-regulated following nusinersen treatment, a change that was statistically significant only in responders in a larger sample [26]. Lower baseline levels of CTSD were significantly associated with a positive treatment response [26]. In another study of 10 adult SMA patients, not one single protein variation was associated with a change in HFMSE after 10 months of treatment with Nusinersen [27]. Nevertheless, using principal component analysis, the authors identified a CSF profile of five differentially affected proteins (NPTX1, SEMA7A, CPE, COL6A1, CDH18) that were collectively associated with treatment response [27]. In a study of 10 SMA type 1 patients, Bianchi et al. showed a general reversion trend of the proteomic pattern to one more similar to normal controls after 6 months of nusinersen treatment [59]. The authors also noted an upregulation of apolipoprotein A1, apolipoprotein E, and transthyretin, which are multifunctional proteins involved in several critical pathways, including synaptogenesis and neurite growth, neuronal survival and plasticity, inflammation, and oxidative stress control [59]. De Wel et al. studied 16 patients with SMA types 3 or 4 in a targeted study of 4 CSF proteins; they found that chitinase-3-like protein 1 (YKL-40) decreased significantly in patients with improvement in RULM [60]. At the time of publication of this study, one additional study of CSF proteomics and metabolomics in 10 SMA type 3 patients had just identified that alterations in SEMA6A, COL1A2, and GRIA4 CSF concentrations at 22 months were associated with HFMSE change [61].

Compared to previous studies, our study used a larger sample of 49 patients, spanning a heterogeneous range of ages, SMA types, and functional levels as seen in clinical practice. In addition, we used an exploratory approach by analyzing 1113 proteins through Olink’s proximity extension assay to identify unsuspected therapeutic pathways and novel proteins that could predict motor improvement after nusinersen; only a few other studies have taken such a wide exploratory approach [26,27,58,61]. It is interesting that, except NEFL, none of the previously reported potential biomarkers of treatment response were identified within the four top proteins most predictive of motor improvement in our sample. Nevertheless, CTSD, which was reported by Schorling et al. to be associated with motor response in pediatric patients [26], was within the 43 proteins whose concentration was significantly reduced at T6 in our cohort. SEMA7A and CPE were also within the 43 proteins significantly reduced after nusinersen treatment in our patients, and these proteins were previously found by Kessler et al. to be associated with treatment response in combination with other proteins [27]. These inconsistencies between proteomic studies may result from different patient populations, differences between detection methods, and lack of standardization in classifying motor responses. The Olink platform uses a proximity extension assay, which is a different approach than the mass spectrometry technique used by others [26,27,61]. The benefits of the proximity extension assay include high sensitivity and low CSF volume requirement, but the main limitation is that the detection is limited to the peptides covered by the panel (for a comparison of different proteomic assays in CSF, see [62]).

Regarding the classification of motor outcomes, other studies have focused on a single functional scale to define motor response, sometimes with varying results within the same study as patients may show improvement on one scale but not another [26,61]. This highlights the complexity of motor response evaluation in SMA patients. In this study, we designed a motor improvement algorithm using published literature on the different scales in order to provide a comprehensive assessment of motor improvement based on all available scales and clinical exams. This allowed us to prioritize the most clinically relevant scale based on the patient’s functional level and age, and to overcome the limitations inherent to using a single scale or time point. However, because most clinical scales for non-sitters have been validated in pediatric populations, many adults in our study did not meet published clinically meaningful difference cut-offs for motor improvement or regression. In order to distinguish those that had some motor improvement within those relatively stable patients, we used natural history data to identify patients that veered from their expected untreated course, which has inherent limitations given the heterogeneity of the published natural history studies and the intrinsic limitations of the scales.

A surprising finding in our results was that patients who functionally improved had numerically less of a decrease in ARSB, ENTPD2, NEFL, IFI30, and CTSD concentrations than did those who did not improve, although these differences were not statistically significant. In the study by Schorling et al., baseline levels of CTSD were significantly lower at baseline in the motor responders, but the decline in both groups was comparable between day 1 to day 300, although it was statistically significant only in responders [26]. This finding remains of uncertain significance and may reflect the limitations of measuring CSF proteomic concentrations, which are likely altered by complex interactions, notably (1) up- or down-regulation of the protein at the cellular level, and (2) neuronal breakdown and leakage into the CSF. Hence, one can postulate a scenario where a given protein may be upregulated at the neuronal level, but where reduced motor neuron breakdown leads to reduced protein leakage into the CSF, leading to paradoxical CSF concentration changes. Moreover, healthier motor neurons may respond better functionally, but contribute less to a change in CSF protein concentrations, allowing for even more complex interactions. This complexity of CSF proteomics is well exemplified in Alzheimer’s disease literature, where amyloid β-42, one of the drivers of Alzheimer’s neuropathology, is paradoxically decreased in the CSF of patients due to brain deposition [63]. Future larger studies are needed to better understand the cellular-level proteomic changes following nusinersen treatment and to explore subgroup effects on proteomic changes according to SMA type and age. Because individual protein concentration changes for the top predictive proteins were not statistically significant between improvers and non-improvers, subgroup analyses were not performed. In future studies, using more than two time points as well as replicates may allow a more confident assessment of trends and reduce biological noise.

A strength of this study was the use of early proteomic changes at 6 months to predict motor improvement at 2 years, which has relevant clinical applications. Indeed, as three SMN-restoring therapies are now available, patients and clinicians are faced with unprecedented therapeutic dilemmas as to which medication to start and how long to continue before concluding that additional treatment is needed. Hence, the prospect of using CSF data for 6 months to help guide ongoing management has broad potential. Our machine learning algorithm was able to predict motor improvement at 2 years with a sensitivity of 80.6% and specificity of 77.8%, showing robust capacity to distinguish between the patients likely to show significant functional benefit. While the individual proteins selected by the model did not exhibit statistically significant differences in their CSF concentration change at T6 between improvers and non-improvers, this does not invalidate their discriminative power in the context of the predictive model. The random forest machine learning algorithm identifies combinations of proteins that are collectively informative for class differentiation. It is likely that prediction of motor improvement cannot rely on the variation of a single protein, but rather on a collection of proteomic changes, as also noted in a prior study [27]. The use of a machine learning data-driven approach leveraged the ability of the random forest to capture these complex nonlinear relationships and provided a robust and objective criterion for feature selection. A limitation of the current approach was that the predictive ability of the model was tested on the same data set used for derivation, which can lead to overfitting. Validation on independent and larger datasets is required to confirm the results presented here and could enhance the model’s potential applicability in clinical settings. The use of alternative methods of protein measurements could also strengthen the conclusions. Finally, studying larger diverse cohorts would facilitate a better understanding of different responses in pediatric vs. adult populations, and in patients with lower functional levels, where significant floor effects impact analysis of currently validated motor outcome measures.

## 5. Conclusions

This exploratory CSF proteomic study of SMA patients spanning a wide range of ages, SMA types, and functional levels seen in neuromuscular clinics showed that most proteins had a reduction in CSF concentration after 6 months of nusinersen treatment. A total of 43 proteins showed a statistically significant change in concentration, of which ARSB, ENTPD2, NEFL, and IFI30 were selected by a machine learning algorithm to predict motor improvement at 2 years. The use of machine learning predictive models that can integrate biomarker and clinical data is a promising avenue to guide treatment decisions in SMA and to optimize individual patient outcomes. Validation in larger, independent, and diverse datasets will enhance the model’s predictive ability and help confirm the results presented here.

## Figures and Tables

**Table 1:** Demographic and baseline clinical characteristics of the SMA patients included in this study.

SMA Type	Type 1 (*n* = 10)	Type 2 (*n* = 18)	Type 3 (*n* = 21)	Total (*n* = 49)
Age (median, range)	9 m (3 m–15 y)	17 y (1 y–50 y)	32 y (1 y–65 y)	18 y (3 m–65 y)
Disease duration at treatment initiation in years (median, range)	0.7 (0.2–15.4)	16.4 (1.0–50.2)	26.7 (1.0–50.6)	17.5 (0.2–50.6)
Gender (M/F)	3/7	10/8	14/7	27/22
*SMN2* copy numbers * (*n*, *%*)				
2 *SMN2*	8 (100%)	0	1 (5%) *	9 (18%)
3 *SMN2*	0	16 (89%)	11 (53%) *	27 (55%)
≥4 *SMN2*	0	0	9 (43%)	9 (18%)
Unknown	2	2 ^†^	0	4 ^†^ (8%)
Functional status				
Infants < 12 mo	6 (60%)	0	0	6 (12%)
Non-sitters	4 (40%)	7 (39%)	1 (5%)	12 (24%)
Sitters	0	11 (61%)	11 (53%)	22 (45%)
Walkers	0	0	9 (43%)	9 (18%)
Highest motor milestone				
None	10 (100%)	0	0	10 (20%)
Sitting	0	18 (100%)	0	18 (37%)
Walking	0	0	21 (100%)	21 (43%)
Use of non-invasive ventilation (*n*, *%*)	9 (90%)	10 (56%)	1 (5%)	20 (41%)
Enteral tube (*n*, *%*)	9 (90%)	2 (11%)	0	11 (22%)
Motor function at 2 years of treatment (*n*, *%*)				
Functional improvement	8 (80%)	10 (56%)	13 (62%)	31 (63%)
No definite functional improvement	2 (20%)	8 (44%)	8 (38%)	18 (37%)

m = months, y = years. * Note that *SMN2* copy numbers may reflect “greater or equal to” the number shown, as some clinical labs reported “at least 2” or “at least 3” copies. ^†^ One patient had 1 *SMN2* copy and a compound heterozygous point mutation on the single SMN1 allele.

**Table 2:** Results on standardized motor function scales at baseline and after 2 years of treatment for SMA patients who either showed functional improvement or did not show functional improvement.

Score Median (Q1, Q3)	Full Cohort (*n* = 49)	Improvers (*n* = 31)	Non-Improvers (*n* = 18)
CHOP-INTEND	*n =* 25	*n =* 18	*n =* 7
Baseline	23 (10, 29)	23.5 (14, 28.75)	10 (9, 38.5)
After 2 years	30 (8, 38)	30.5 (18.25, 37.5)	8 (5.5, 33)
Median change	+3 (−1, +6)	+5 (+2.25, +10.5)	−2 (−4.5, −1)
HFMSE	*n =* 17	*n =* 10	*n =* 7
Baseline	19 (8, 40)	11 (3.25, 29.5)	40 (17, 51)
After 2 years	15 (6, 38)	13 (6, 34)	38 (12, 42.5)
Median change	+2 (−4, +3)	+3 (+2, +3.75)	−5 (−6, −3.5)
RULM	*n =* 31	*n =* 19	*n =* 12
Baseline	18 (8.5, 33.5)	15 (8.5, 23.5)	34 (13.25, 37)
After 2 years	20 (10, 33.5)	16 (10, 23.5)	31.5 (8.75, 34.25)
Median change	0 (−0.5, +2)	+1 (0, +2)	−1 (−3.25, 0)
6MWT	*n =* 8	*n =* 2	*n =* 6
Baseline	321.5 (270.75, 401.5)	429.5 (411.25, 447.75)	301.5 (240.25, 323.75)
After 2 years	284.5 (237.75, 401.5)	483.5 (482.25, 484.75)	266 (193.25, 289.25)
Median change	−26 (−51.25, −10)	+54 (+37, +71)	−40 (−51.75, −24.5)

CHOP-INTEND: Children’s Hospital of Philadelphia Infant Test of Neuromuscular Disorders; HFMSE: Hammersmith Functional Motor Scale Expanded; RULM: Revised Upper Limb Module; 6MWT: 6-Minute Walking Test.

## Data Availability

The datasets generated and/or analyzed during the current study are not publicly available given that they contain private health information and consent was obtained to provide only cumulative anonymized data. A minimal dataset is available from the corresponding author upon reasonable request.

## Supplementary Materials

The following supporting information can be downloaded at: https://www.mdpi.com/article/10.3390/jcm12206696/s1, Table S1: list of the 43 proteins with statistically significant CSF concentration changes between baseline and 6 months in the whole cohort.; Figure S1: individual patient-level changes in each of the five identified proteins between T0 and T6.

## Appendix A. Motor Improvement Evaluation Algorithm

The following motor functional scales were used with their respective minimal clinically important difference (MCID) or, if unavailable, distribution-based minimal detectable change (MDC). For each SMA type, functional level, and age group, the most relevant and discriminative clinical scale was defined as first intention, and then second, third, and fourth intention scales were defined, to address situations where some scales were not performed or where the results from higher intention scales fell into a grey zone (small variation not meeting MCID/MDC cut-offs). Relevant natural history studies are discussed to support the choice of cut-offs. The supporting literature is referred to in brackets.

**Scale**	**Improver**	**Non-Improver**
HFMSE [30,31]	≥+2	≤−2
CHOP-INTEND [10]	≥+4	≤−4
RULM [32]	≥+2	≤−2
6MWT [33,34]	≥+30 m	≤−30 m

(1) SMA type 1
  (a) First intention scale: CHOP-INTEND.
  (b) Second intention scale: RULM.
  (c) Use MCID/MDC cut-offs. If patient falls in grey zone, consider other scales or rate of change. Rate of change was calculated with a linear model using all available time points up to the two-year assessment. Any stability (rate of change = 0) or positive rate of change is considered to be an “improver” since accumulating evidence suggests that stabilization or minimal gain is viewed as clinically significant by SMA patients and caregivers [39] and defies the natural history in SMA type 1 patients [37,38]. Any negative rate of change is a “non-improver”.
  (d) Natural history data: In the control group from the ENDEAR trial, no infant improved on the HINE-2 scale, and only 1 in 41 improved on the CHOP-INTEND [10]. In one study, the rate of decline on CHOP-INTEND in untreated patients was −1.27 points/year (95%CI −2.33 to −0.21) [8].
(2) SMA type 2
  (a) First intention scale: HFMSE.
  (b) Second intention scale: RULM.
  (c) Third intention scale: CHOP-INTEND (CHOP-INTEND was used as the first scale for all patients < 24 months old).
  (d) HFMSE is prioritized for all type 2 patients since it is largely validated, has established minimal clinically important differences [31], and the magnitude of observable changes is usually larger than in RULM, allowing for an easier evaluation of clinical improvement. Moreover, the trajectory of untreated patients was more clearly negative using HFMSE in a systematic review, which allows for a better identification of responders [12]. However, in patients with minimal change or minimal positive change on HFMSE, RULM may be able to capture improvement in more severely affected patients that rely mainly on upper extremity function [45].
  (e) If <5 y.o.:
    (i) Use MCID/MDC cut-offs. If patient falls in grey zone, consider other scales or rate of change, using all available time points up to the two-year assessment. Any positive rate of change in HFMSE is considered an “improver”, any stability (rate of change = 0) or negative change is a “non-improver”. In RULM, any positive rate of change above 0.9 point/y is considered an “improver”; anything below that value is categorized as a “non-improver”.
    (ii) Natural history data shows that patients may improve on HFMSE in that age group [40,41,45]. Nevertheless, in the largest study focusing only on type 2 patients and reporting changes at 24 months, the mean change in HFMSE was −0.51 in that specific age group [42]. In another natural history study, the mean 12-month change was +0.04 (SE = 0.34) in a group pooling type 2 and type 3 non-ambulant patients [41]. The placebo arm from the SUNFISH part 2 trial had an increase of +2.59 points/year, but this group also pooled type 2 and type 3 non-ambulant patients, which are more likely to improve early on [45]. Because of the heterogeneous natural history fluctuations in HFMSE reported by different studies, the authors decided to use primarily the only study that addressed specifically type 2 patients, which reported a negative change in HFMSE. In order to adopt a conservative approach that takes into account the different individual trajectories, stabilization was considered an “improver” in that specific age group. In RULM, SMA type 2 patients show a mean increase of 0.9 points (SD 4.2) over 12 months in that age group [32].
  (f) If ≥5 y.o.:
    (i) Use MCID/MDC cut-offs. If patient falls in grey zone, consider other scales or rate of change, using all available time points up to the two-year assessment. Any stabilization or positive rate of change is considered an “improver”, any negative change is a “non-improver”.
    (ii) Natural history and placebo arm from the CHERISH trial show clear deterioration in HFMSE in the age group between 5 and 13 y.o., estimated at −0.96 and −2.15 points/year [11,40,41]. After 13 y.o., patients will show a slow decline in HFMSE, most noticeable at 24 and 36 months [40,43,44,45]. In RULM, patients have a mean decline of −1.52 points (SD 2.91) over 24 months after 5 years of age [46].
(3) SMA type 3: walker
  (a) First intention scale: 6MWT.
  (b) Second intention scale: HFMSE (HFMSE was used as first scale for patients between 24m and 36m of age).
  (c) Third intention scale: RULM.
  (d) Fourth intention scale: CHOP-INTEND (CHOP-INTEND was used as first scale for all patients < 24 months old).
  (e) Note: 6MWT is prioritized over HFMSE since it discriminates between disease severity, is sensitive to fatigue-related changes, and is responsive to longitudinal gains in post hoc analyses of clinical trials [34,35,36].
  (f) If <7 y.o.:
    (i) Use MCID/MDC cut-offs. If patient falls in grey zone, consider other scales or rate of change, using all available time points up to the two-year assessment. Any positive rate of change in HFMSE > +1.51 point/y is considered an “improver”, any rate of change below that would be a “non-improver”. Any positive change in RULM > 1.8 point/y is considered an “improver”, any rate below that would be a “non-improver”.
    (ii) Natural history studies have shown that young patients (<6 y.o.) improve on 6MWT by a mean of 9.8 m/y [47]. This is below the minimal detectable change cut-off, so one can be confident that improvement ≥30 m is above what would be expected according to natural history. Before 7 years of age, patients significantly improve on HFMSE at a rate of +1.51/y (0.82–2.19), according to a multivariate model up to a breaking point at 7 years old, after which most patients either decline or stabilize [30]. In RULM, ambulant type 3 SMA patients improved by a mean of 1.8 points (SD 5.8) before 5 years of age [32].
  (g) If ≥7 y.o.:
    (i) Use MCID/MDC as a cut-off for both “improvers” and ”non-improvers” [34]. If patient falls in grey zone, look at other scales or rate of change, using all available time points up to the 2y assessment. Any stabilization (rate of change = 0) or positive rate of change in 6MWT or HFMSE is considered an “improver”, any negative change is a “non-improver”. For RULM, a rate of change > +0.20 point/y was considered an “improver”, and anything below that a “non-improver”.
    (ii) One longitudinal natural history study showed that no patient improved in HFMSE after the age of 7 years [30,48]. Similarly, 6MWT was shown to decline continuously in patients older than 6 years of age with mean annual changes between −7.9 point/y and −20.8 m/y, with the most rapid decline between 11 and 19 years of age [47]. Mean pooled decrease in HFMSE over one or two years of treatment in a recent systematic review was −1.00 (95% CI−1.33; −0.67) for patients with SMA types 2 and 3 over all ages, although there was considerable heterogeneity. In RULM, type 3 ambulant patients show a mean increase of 0.40 points (SD 1.87) over 24 months [46], so a rate of change >+0.20 point/y was calculated for ”improvers”.
(4) SMA type 3: non-walkers at beginning of study
  (a) First intention scale: HFMSE.
  (b) Second intention scale: RULM.
  (c) Third intention scale: CHOP-INTEND (CHOP-INTEND used as first scale for all patients < 24 months old).
  (d) If <7 y.o.:
    (i) Use MCID/MDC cut-offs. If patient falls in grey zone, look at other scales or rate of change, using all available time points up to the 2 y assessment. Any positive rate of change in HFMSE above +1.51 point/year would be considered responder, any rate of change below that would be non-responder.
    (ii) Natural history data and data from placebo groups in SUNFISH part 2 show that patients can improve on HFMSE in that age group, as discussed previously [30,41,45]. Before 7 years of age, patients significantly improve on HFMSE at a rate of +1.51/y (0.82–2.19), according to a multivariate model up to a breaking point at 7 years old, after which most patients either decline or stabilize [30]. No specific data is available for RULM for that age group [32].
  (e) If ≥7 y.o.:
    (i) Use MCID/MDC cut-offs. If patient falls in grey zone, look at other scales or rate of change, using all available time points up to the 2 y assessment. Any stability or positive rate of change in HFMSE or RULM is considered an “improver”, and any negative change is a “non-improver”.
    (ii) Natural history studies and placebo arm from SUNFISH part 2 showed that in older patients with non-ambulant type 3 SMA, the HFMSE and RULM can be stable over a 12-month period, although patients will eventually show a slow decline in HFMSE at 24 and 36 months [40,43,44,45]. In that age group, natural history is characterized by a very slow deterioration. For RULM, the mean 24-month change was −0.94 points (SD 2.67) [46].
