# Supplementary material for: Cerebrospinal Fluid Proteomic Changes after Nusinersen in Patients with Spinal Muscular Atrophy
Source: J Clin Med. 2023 Oct 23;12(20):6696. doi: 10.3390/jcm12206696 (PMC10607664; doi:10.3390/jcm12206696)

SUPPLEMENTAL DATA

Table S1. List of the 43 proteins with statistically significant CSF concentration changes between baseline and 6 months in the whole cohort. Proteins are ordered in alphabetical order.

Protein Name	NPX Baseline	NPX 6 Months	p-value
**5'-NT**	5.711922	5.381071	0.037223
**ARSA**	4.735977	4.267065	0.000504
**ARSB**	2.361769	1.872142	0.000017
**BCAN**	8.673662	8.442559	0.001875
**CD164**	8.232515	7.939035	0.016380
**CD200**	10.669911	10.152285	0.019400
**CDCP1**	2.720409	2.316968	0.001386
**CLSTN2**	10.854361	10.494110	0.008050
**CNTNAP2**	8.382700	7.912044	0.006442
**CPE**	7.993343	7.419813	0.006202
**CTSD**	5.826079	5.623149	0.039212
**CTSH**	3.469933	2.975400	0.018446
**CTSO**	6.632594	5.974313	0.000683
**CTSV**	2.001149	1.748090	0.023094
**CTSZ**	5.307407	5.001827	0.003184
**DDR1**	7.420804	6.918903	0.008615
**DPP7**	7.491654	6.970284	0.000253
**ENTPD2**	3.391056	2.823861	0.000016
**ERBB4**	10.491324	10.159281	0.041420
**FUCA1**	6.560719	6.302826	0.032307
**GPC1**	2.355918	1.873136	0.012237
**GUSB**	3.098409	2.821535	0.011704
**IFI30**	1.279295	0.883697	0.004786
**IGSF3**	3.717674	3.354756	0.009366
**IL17RB**	3.243106	2.903834	0.007429
**LRIG1**	5.800243	5.203965	0.000788

**MARCO**	2.720275	2.258831	0.005611
**MDGA1**	4.437453	3.829840	0.012666
**N2DL-2**	4.456201	3.806864	0.003843
**NEFL**	7.852687	6.716491	0.001734
**NEP**	0.562517	0.395756	0.012042
**PLXNA4**	3.242533	2.860491	0.034911
**RET**	3.639689	3.294613	0.016265
**ROBO2**	5.222094	4.775604	0.037431
**RTN4R**	5.639478	5.236186	0.004936
**SEMA7A**	9.212509	8.657786	0.011994
**SMPD1**	1.501108	1.080332	0.026106
**SUMF2**	5.917254	5.646664	0.039325
**TLR3**	4.776156	4.359870	0.040837
**TMPRSS5**	5.896337	5.429499	0.040542
**TN-R**	6.365707	6.156418	0.041959
**TPP1**	10.119076	9.605972	0.000295
**WIF-1**	6.233285	5.618720	0.029001

Figure S1. Individual patient-level changes in each of the five identified proteins between T0 and T6. Y axis represents NPX values and x axis is individual patient number. Data is shown for motor improvers on the left (a) to (e) compared with non-improvers on the right (f) to (j). Each row represents one of the selected proteins with (a) and (f) ARSB, (b) and (g) ENTPD2, (c) and (h) NEFL, (d) and (i) IFI30, and (e) and (j) CTSD. Patients are ordered from youngest to oldest at T0. Only NEFL in (c) and (h) appeared to show an effect of age on baseline concentration and post-treatment change.

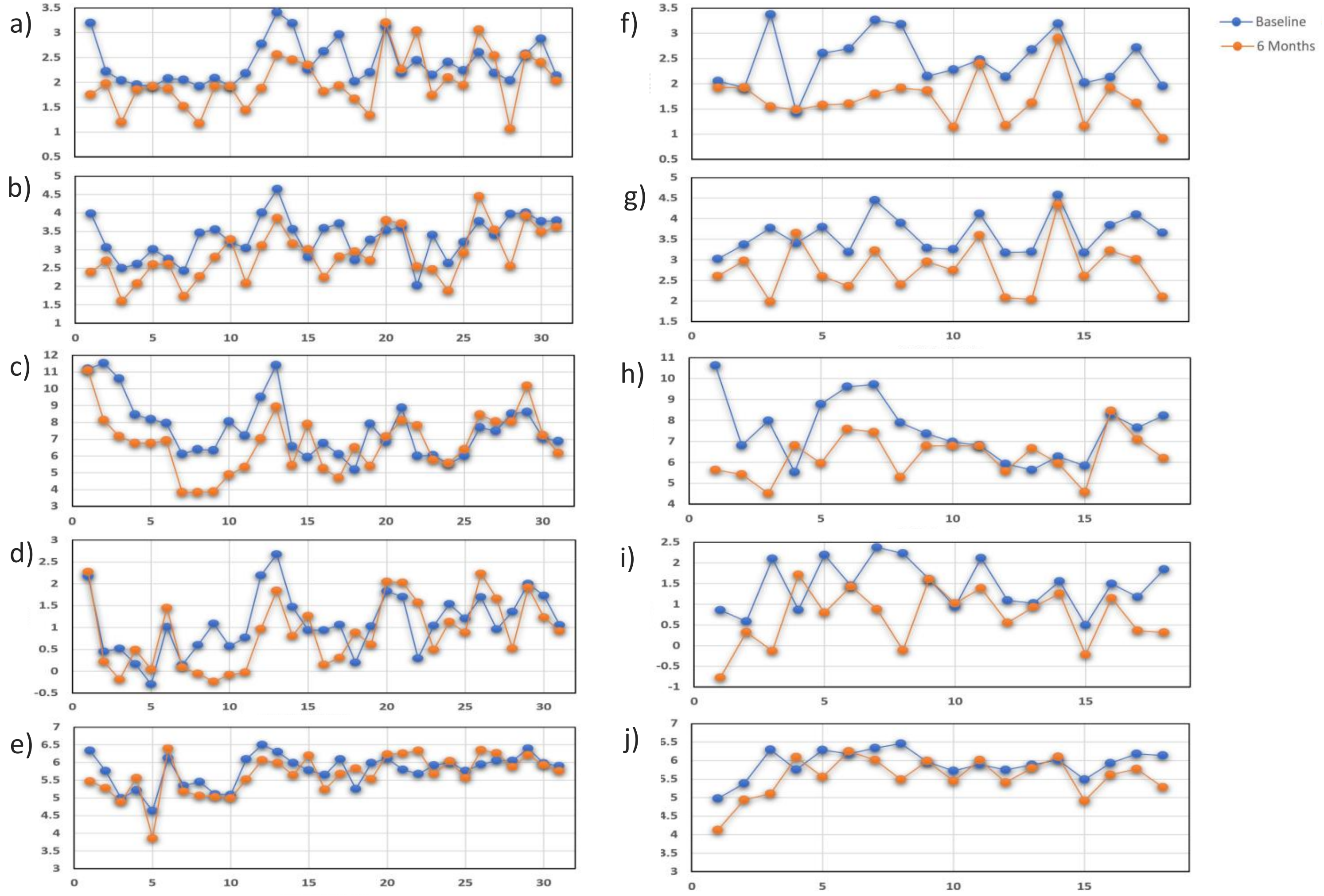

Supplement: Supplementary file 1 [file jcm-12-06696-s001.zip › jcm-2617230-supplementary.pdf]
